# Effect of Hybrid Fiber on the Chloride Salt Erosion Resistance of Shotcrete

**DOI:** 10.3390/ma19071352

**Published:** 2026-03-29

**Authors:** Peng Hu, Hongyu Ji, Baicheng Liu, Kun Wang, Song Han, Fuying Dong, Yulong Zhao

**Affiliations:** 1 School of Transportation and Civil Engineering, Shandong Jiaotong University, Jinan 250357, China; 204021@sdjtu.edu.cn (P.H.); 23107013@stu.sdjtu.edu.cn (H.J.); 23107023@stu.sdjtu.edu.cn (B.L.); wangkun@sdjtu.edu.cn (K.W.); dongfuying2000@aliyun.com (F.D.); 2 Shandong Key Laboratory of Technologies and Systems for Intelligent Construction Equipment, Jinan 250357, China; 3 Shandong Key Laboratory of Intelligent Construction and Operation of Highway Infrastructure, Jinan 250014, China; 4 Jinan City Investment Design Co., Ltd., Jinan 250101, China; hs22107009@126.com

**Keywords:** hybrid fiber, shotcrete, chloride erosion, mechanical properties, durability

## Abstract

**Highlights:**

- The shotcrete mix ratios were optimized using PF and BF.
- Immersion and salt spray tests simulated chloride erosion in ocean environments.
- Shotcrete with 0.2% PF and 0.1% BF showed optimal resistance to chloride erosion.
- The chloride erosion mechanism was analyzed at fine and microscopic scales.

**Abstract:**

The use of shotcrete is a critical support technique in ocean engineering structures. However, it often exhibits low chloride and salt erosion resistance under ocean environmental conditions and poor long-term durability. This study employed polypropylene fiber (PF) and basalt fiber (BF) to optimize the shotcrete mix design. Laboratory immersion and salt spray tests simulated chloride ion corrosion environments in the ocean’s underwater and atmospheric zones. The effects of different corrosion mechanisms and varying fiber volume fractions on shotcrete strength and durability were then analyzed. The results indicate that shotcrete demonstrates strong resistance to chloride-induced corrosion in both ocean underwater and atmospheric zones when the volume fractions of PF and BF are 0.2% and 0.1%, respectively. Based on test results from 3D digital microscopy (3D-DM), X-ray diffraction (XRD), and scanning electron microscopy (SEM), the chloride-induced degradation mechanism of hybrid fiber-reinforced shotcrete was analyzed from both mesoscopic and microscopic perspectives. This study offers theoretical support for applying hybrid fiber-reinforced shotcrete in ocean engineering environments.

## 1. Introduction

Shotcrete is a construction technique in which fresh concrete is pneumatically projected onto a substrate using compressed air [1,2,3,4]. Shotcrete offers advantages such as high early strength, broad applicability, and efficient construction, making it widely used in tunnel linings, slope stabilization, and underground engineering projects [5,6]. With the rapid expansion of global ocean resource exploitation and coastal urban development, shotcrete is being increasingly applied in structures such as submarine tunnels, port terminals, and coastal dams [7,8,9]. However, shotcrete structures in ocean environments are subjected to prolonged chemical erosion by chlorides, sulfides, and carbonates, with chloride-induced degradation particularly prominent [10,11,12,13]. Chloride-induced erosion can lead to cracking of shotcrete, corrosion of reinforcing steel, and strength degradation, thereby posing a direct threat to ocean infrastructure’s service life and structural safety [14,15,16,17]. Enhancing the resistance of shotcrete to chloride-induced erosion has become a pressing challenge in ocean engineering construction.

Currently, most research focuses on enhancing shotcrete’s chloride and sulfate resistance through mineral admixtures and chemical additives. For example, Sakoparnig et al. [18] optimized the shotcrete mix design using fly ash and limestone. Their results indicated that mineral admixtures’ hydration enhances shotcrete’s chloride ion penetration resistance by refining its microstructure and increasing its chloride-binding capacity. Teymouri et al. [19] and Yang et al. [20] reported that the pozzolanic reaction and secondary hydration of fly ash and silica fume enhance the compactness of shotcrete, thereby significantly improving its resistance to chloride ion penetration. Li et al. [21] investigated the influence of mineral admixtures on concrete’s resistance to sulfate and chloride erosion, concluding that incorporating 15% limestone powder reduces pore formation through filling and nucleation effects, hinders chloride migration and infiltration, and improves the strength and durability of concrete. Yang et al. [22] and Wang et al. [23] analyzed the effects of various accelerators on shotcrete’s resistance to sulfate and chloride corrosion and found that high dosages of accelerators negatively impact concrete durability. Garba et al. [24,25] systematically reviewed the effects of accelerators on the long-term performance of shotcrete and compared the performance of different types in resisting sulfate erosion and calcium dissolution. They concluded that alkali-free fluorinated accelerators provided the highest resistance to sulfate erosion. Although appropriate amounts of mineral admixtures and alkali-free accelerators enhance shotcrete’s chloride ion erosion resistance, they also significantly prolong its setting time, which hinders the development of early strength.

Fiber-reinforced shotcrete has garnered significant attention recently due to its superior mechanical properties and durability [26]. Considerable progress has been made in applying single fibers in shotcrete, with steel fiber-reinforced shotcrete being the most widely used [27,28,29]. For example, Zhang et al. [30] and Liu et al. [31] investigated the effect of steel fibers on shotcrete performance, concluding that steel fibers enhance shotcrete strength and toughness. Manquehual et al. [32] investigated the service performance of steel fiber-reinforced shotcrete in an undersea tunnel. They found that leaching increased porosity, reducing the material’s density and strength. Although steel fibers can significantly enhance the strength of shotcrete, they are susceptible to corrosion, exhibit poor dispersion, and involve high construction costs [33,34,35]. As a result, the use of non-metallic fibers in shotcrete has increasingly attracted research attention. For example, Khooshechin et al. [36] modified glass fibers with nanomaterials to enhance the flexibility of shotcrete and effectively inhibit crack development. Song et al. [37] and Guo et al. [38] concluded that BF significantly improves shotcrete’s flexural strength, fracture toughness, and freeze–thaw resistance. Alomayri et al. [39] concluded that PF helps control tidal erosion in concrete, with a 0.15% fiber content reducing mass loss by 0.34%. The studies above demonstrate that fibers significantly enhance shotcrete’s strength, fracture toughness, and freeze–thaw resistance. However, the performance of different fiber types varies considerably, and single fibers have inherent limitations in improving overall shotcrete performance. As a new type of composite material, hybrid fiber shotcrete has gradually attracted increasing attention. It typically consists of two or more fibers with different properties, sizes, or materials combined in specific proportions to produce a synergistic effect and overcome the limitations of single-fiber reinforcement [40,41]. Some researchers have begun to incorporate hybrid fibers into shotcrete. For example, Zhao et al. [42] and Jawhar et al. [43] investigated the effects of polypropylene fibers and waste plastic fibers on shotcrete performance. Their results indicated that hybrid fiber-reinforced shotcrete exhibited superior mechanical properties to single-fiber shotcrete. Hu et al. [44,45] and Zheng et al. [46] investigated the effects of single and hybrid alkali-resistant glass fibers on the mechanical and microstructural properties of shotcrete and ultra-high-performance concrete. Their results showed that, under hybrid fiber conditions, alkali-resistant glass fibers significantly enhanced concrete strength and improved its microstructure. Mehmandari et al. [47] investigated hybrid fiber shotcrete’s strength and deformation properties and concluded that it could effectively reduce deformation and tensile damage in surrounding rock. Although research on hybrid fiber shotcrete has made significant progress, most studies focus on its early strength and deformation, with fewer investigations on its resistance to chloride and salt erosion. Notably, research on shotcrete in ocean atmospheric environments remains limited. However, chloride salt erosion directly threatens the safe service of shotcrete, highlighting the need for in-depth research on the resistance of hybrid fiber shotcrete to chloride salt erosion.

PF is an organic synthetic fiber characterized by low elastic modulus and high ductility. In cement-based materials, PF inhibits the initiation and development of cracks through physical bridging. The study shows that polypropylene fibers can significantly improve the tensile strength of concrete. However, due to their low modulus of elasticity, their ability to enhance the compressive strength and stiffness of concrete is relatively limited [48,49]. BF is a natural inorganic silicate mineral fiber with high elastic modulus and tensile strength, but relatively low ductility. Studies have shown that BF can significantly improve the compressive strength and deformation resistance of concrete [50,51]. Based on the performance characteristics of the two fibers mentioned above, this paper proposes to combine PF and BF in shotcrete to leverage their synergistic effect, compensate for the shortcomings of single fibers in improving concrete performance, and thus improve the chloride erosion resistance of shotcrete.

This paper aims to enhance shotcrete’s resistance to chloride salt erosion and ensure the safe service of ocean engineering structures. To achieve this, the shotcrete mix was optimized using PF and BF. The chloride ion erosion environments of the underwater ocean and ocean atmospheric areas were simulated through immersion and salt spray tests. The effects of different erosion modes on shotcrete’s mechanical properties and durability were then analyzed. The deterioration mechanism of chloride ion erosion in hybrid fiber shotcrete was analyzed based on fine and microscopic tests. This study aims to contribute to applying and developing hybrid fiber shotcrete in ocean engineering.

## 2. Materials and Test Methods

### 2.1. Materials and Mix Ratio

#### 2.1.1. Materials

The cement used is P•O 42.5 grade cement produced by Shandong Shanshui Cement Group Co., Ltd. (Jinan, China), with all indices meeting the requirements of “Common portland cement” (GB175-2023) [52]. The main chemical components of cement are shown in Table 1. The coarse aggregate used is continuously graded gravel with a particle size of 5–10 mm, while the fine aggregate is natural river sand with a fineness modulus of 2.75. All aggregate properties meet the requirements of the “Technical code for shotcrete application” (JGJ/T372-2016) [53]. The admixtures used include a polycarboxylic acid-based high-efficiency superplasticizer and a liquid, alkali-free, fluorine-free accelerator. All indicators comply with the “Code for utility technical of concrete admixture” (GB 50119-2013) [54]. Basalt fibers and polypropylene fibers were selected, and their main performance parameters are listed in Table 2.

#### 2.1.2. Shotcrete Mix Ratio

The initial mix ratio of shotcrete was determined according to the “Specification for mix proportion design of ordinary concrete” (JGJ55-2011) [55] and the “Technical code for shotcrete application” (JGJ/T 372-2016) [53]. After laboratory mix testing, trial spraying, and subsequent adjustments, the final base mix proportion of 1 m^3^ shotcrete was determined as cement: gravel: sand: water = 450 kg:864 kg: 864 kg:198 kg. The superplasticizer and accelerator agent dosages were 0.8% and 6.0% of the cementitious material mass, respectively. The specification “Technical specification for application of fiber reinforced concrete” (JGJ/T221-2010) requires that the volume fraction of fiber in shotcrete be in the range of 0.06~0.25% [56]. Current research indicates that excessive fiber volume fractions promote fiber agglomeration in concrete, thereby adversely affecting its workability [57,58]. Therefore, the total content of blended fibers in this study was limited to no more than 0.4%, and the corresponding fiber combinations are presented in Table 3.

### 2.2. Test Methods

#### 2.2.1. Specimen Preparation Method

**Figure 1:**
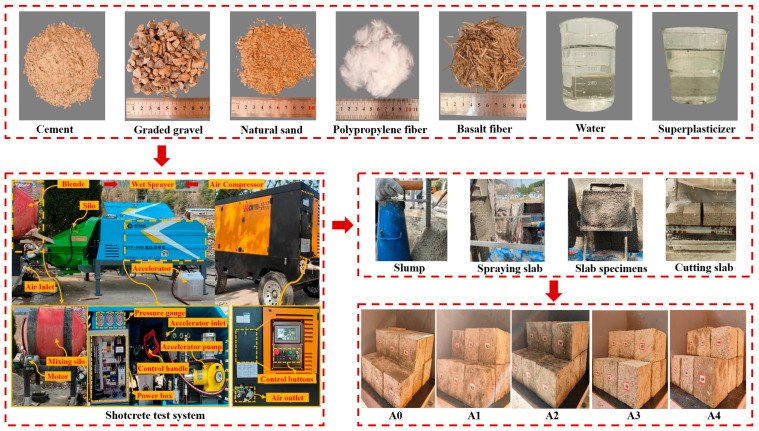
Specimen preparation process.

Concrete specimens were prepared by spraying the concrete onto slab molds. The dimensions of the concrete slab molds were 350 mm × 450 mm × 120 mm. Before testing, the concrete slab molds were fixed in the test shed at an angle of 80° to the horizontal. Based on different fiber–concrete mix designs, 400 kg of concrete was prepared for each, and slump tests were conducted. The results are shown in Table 4. During the test, the working wind pressure of the concrete wet sprayer was set to 0.4 MPa, and the nozzle was positioned 1 m away from the slab mold. The hybrid fiber concrete was sprayed vertically into the mold. After the test, the concrete slabs were demolded after 24 h of curing and then transferred to a standard curing room (Temperature 20 ± 2 °C, Relative humidity ≥ 95%) for further curing. After curing to the designated age, the concrete slabs were cut using a rock cutter into cubic specimens (100 mm × 100 mm × 100 mm) and prismatic specimens (100 mm × 100 mm × 400 mm). A total of 300 cubic specimens were used for compressive strength tests, splitting tensile strength tests, and mass loss tests. A total of 150 prismatic specimens were used for flexural strength tests and dynamic modulus of elasticity tests. The specimen preparation process is shown in Figure 1.

#### 2.2.2. Chloride Erosion Test

To investigate the effects of chloride ion erosion on the performance of hybrid fiber shotcrete, two environmental conditions were simulated: the ocean underwater zone and the ocean atmospheric zone. The indoor immersion test simulated the underwater ocean environment using artificial seawater composed of a 5.0% NaCl solution at an ambient temperature of 25 °C. The ocean atmospheric environment was simulated using a salt spray test, following the “Corrosion tests in artificial atmospheres-Salt spray tests” (GB/T 10125-2021) guidelines [59]. The laboratory temperature was set to 35 °C, and the pressure drum temperature was set to 40 °C. Intermittent spraying (4 h followed by 4 h of demisting) was employed to conduct periodic salt spray erosion on the concrete specimens. Indoor immersion and salt spray tests were conducted to evaluate the performance indices of shotcrete after 28 d, 56 d, 84 d, and 112 d of chloride exposure.

#### 2.2.3. Concrete Strength Test

The concrete strength tests were conducted by the “Standard for testing and detecting the physical and mechanical properties of concrete” (GB/T 50081-2019) [60]. A universal testing machine was used to measure the hybrid fiber-reinforced shotcrete’s compressive strength and splitting tensile strength. The specimens for the compressive strength test measured 100 mm × 100 mm × 100 mm. The loading rate was set between 0.3 and 0.5 MPa/s, and a strength conversion factor of 0.95 was applied. The splitting tensile strength test was conducted using a splitting fixture. Specimens measured 100 mm × 100 mm × 100 mm, with a loading rate of 0.02 to 0.05 MPa/s and a strength conversion factor of 0.85. The flexural strength of hybrid fiber shotcrete was tested using a concrete flexural testing machine. The specimens measured 100 mm × 100 mm × 400 mm, with a loading rate of 0.02 to 0.05 MPa/s and a strength conversion factor of 0.85. Take three specimens of different mixing ratios made and maintained at the same age for parallel testing, and take the arithmetic average of the test results of the three specimens. Due to the significant influence of accelerators and the early-stage hydration of cement on the 3 d and 7 d strengths of shotcrete, it is difficult to effectively demonstrate the synergistic strengthening effect of the blended fibers. Therefore, this study adopted a 28 d testing cycle to fully reflect the strength decay patterns of shotcrete under chloride ion attack at different ages.

**Figure 2:**
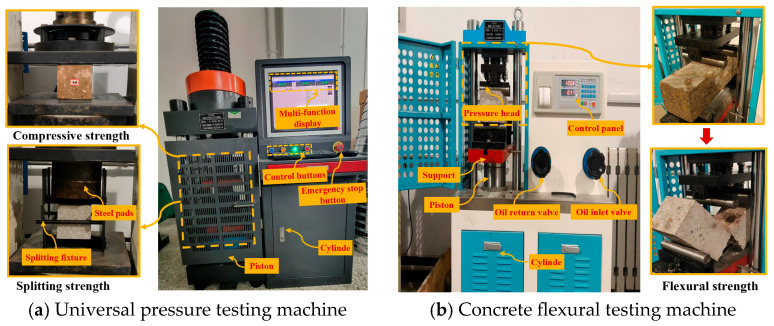
Test loading device.

The test loading device is shown in Figure 2.

#### 2.2.4. Concrete Durability Test

The dynamic elastic modulus and mass loss rate of hybrid fiber shotcrete were tested according to the “Standard for test methods of long-term performance and durability of concrete”(GB/T 50082-2024) [61]. The specimen size was 100 mm × 100 mm × 400 mm, and the vibration frequency range was between 1500 and 3000 Hz. An electronic balance was used to test the mass of mixed fiber shotcrete at different erosion ages, and the test results were taken as the arithmetic mean of three specimens, with results accurate to 0.1%. The KS-X1000 3D-DM was used to observe the microscopic porosity of hybrid fiber shotcrete at different erosion ages, with a resolution of 1 μm and a scanning speed of 20 mm/s.

#### 2.2.5. Microscopic Test

**Figure 3:**
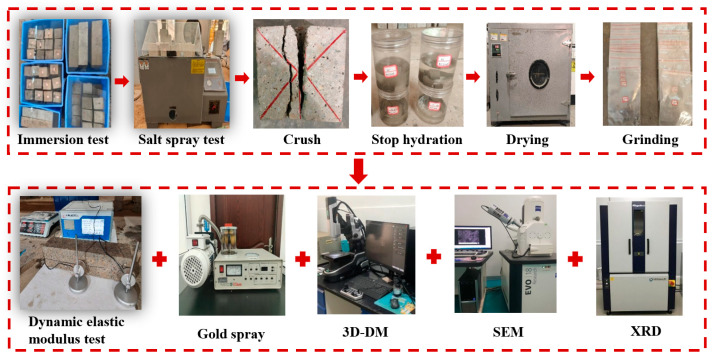
Durability test and microtesting process.

The hydration products and micromorphology of hybrid fiber shotcrete at different erosion ages were analyzed using a Smart Lab XRD (Rigaku, Tokyo, Japan) and a ZEISS Sigma 500 SEM (ZEISS, Jena, Germany). First, the specimens at the designated ages were crushed and immersed in anhydrous ethanol to terminate hydration. Small fragments were then dried in an oven at 60 °C for 24 h and prepared into flakes approximately 5 mm in size and concrete powder with a particle size of 0.075 mm. The XRD test was conducted using a Cu target with a scanning angle of 5° to 80° and a 2°/min scanning rate. Due to the concrete’s poor electrical conductivity, a gold sputter coating was applied to the concrete slices before SEM testing. The process of the shotcrete durability test and microscopy test is shown in Figure 3.

## 3. Results and Discussion

### 3.1. Effects of Different Erosion Environments on the Mechanical Properties of Shotcrete

#### 3.1.1. Compressive Strength Analysis

**Figure 4:**
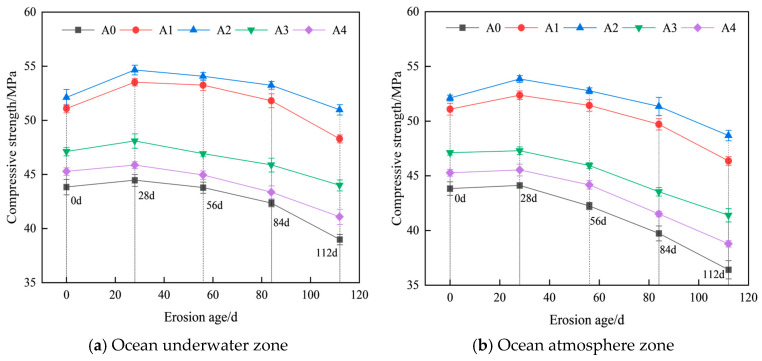
Variation in compressive strength of hybrid fiber shotcrete.

Based on the test results, graphs depicting the changes in compressive strength of hybrid fiber shotcrete at different chloride erosion ages in the ocean underwater and atmospheric zones were plotted (Figure 4).

As shown in Figure 4, the compressive strength of hybrid fiber-reinforced shotcrete is consistently higher than that of conventional shotcrete. Before chloride ion erosion, the 28 d compressive strength of hybrid fiber-reinforced shotcrete increased by 16.56%, 18.89%, 7.48%, and 3.29%, respectively, compared with conventional shotcrete. Among them, 0.2% PF and 0.1% BF hybrids were the most effective in enhancing the compressive strength of shotcrete. This improvement is attributed to the superior dispersion of PF in the shotcrete matrix, which helps inhibit microcrack propagation, and the high elastic modulus of BF, which enhances the shotcrete’s load-bearing capacity. As shown in Figure 4a, the compressive strength of hybrid fiber shotcrete initially increases and then decreases under the chloride salt erosion conditions in the ocean underwater zone. When the age of erosion was 28 d, the compressive strength of hybrid fiber shotcrete was improved by 4.77%, 4.87%, 2.08% and 1.30%, respectively. This can be attributed to the hybrid fibers, which, in the pre-chloride salt erosion stage, inhibit the formation of macrocracks in the shotcrete, thereby reducing the penetration rate of chloride ions and extending the strength development phase. The compressive strength of hybrid fiber shotcrete decreased rapidly after 28 d of chloride salt erosion. When the erosion age was 112 d, the compressive strength of hybrid fiber shotcrete in the ocean underwater zone decreased by 5.48%, 2.19%, 6.62%, and 9.26%, respectively, compared to the pre-erosion period. This is because chloride salts gradually diffuse through the pores into the concrete as the erosion age increases. The chloride ions react with calcium hydroxide and other hydration products to form soluble calcium chloride, damaging the concrete’s internal structure. Simultaneously, sodium ions in the chloride salts can trigger alkali–aggregate reactions in the concrete, leading to expansion and cracking. As shown in Figure 4b, the compressive strength of hybrid fiber shotcrete under chloride salt erosion in the ocean atmospheric zone exhibits a similar trend to that in the ocean underwater zone. However, after 28 d of salt spray erosion, the compressive strength of shotcrete in the ocean atmospheric zone decreased significantly faster than that in the ocean underwater zone. When salt spray erosion was carried out for 112 d, the compressive strength of shotcrete in the ocean atmosphere decreased by 9.27%, 6.58%, 12.14% and 14.34%, respectively, compared with that before erosion. The reason is that salt spray erosion alternates between wet and dry cycles, prompting repeated crystallization of chloride salt in the concrete pores. The resulting expansion stress accelerates the development of concrete microcracks, which significantly accelerates the strength decay of shotcrete.

#### 3.1.2. Splitting Tensile Strength Analysis

**Figure 5:**
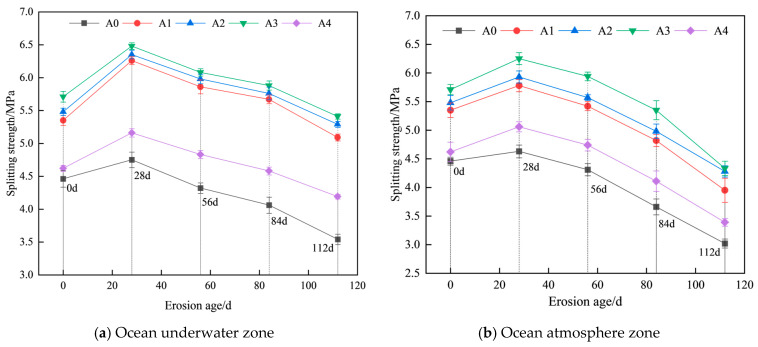
Variation in splitting tensile strength of hybrid fiber shotcrete.

Based on the test results, graphs depicting the variation in splitting tensile strength of hybrid fiber shotcrete with different chloride erosion ages in the ocean underwater and atmosphere zones were plotted (Figure 5).

As shown in Figure 5, hybrid fibers have a more pronounced effect on enhancing shotcrete’s splitting tensile strength than compressive strength. The 28 d splitting tensile strength of hybrid fiber shotcrete before chloride erosion increased by 19.96%, 22.87%, 28.03%, and 3.58%, respectively, compared to conventional shotcrete. Among the tested combinations, the mixture containing 0.1% PF and 0.2% BF exhibited the greatest improvement in shotcrete’s splitting tensile strength. This enhancement is attributed to BF’s high tensile strength, which, when used in appropriate amounts, effectively increases the material’s fracture toughness. As shown in Figure 5a, after 28 d of chloride salt erosion in the ocean underwater zone, the splitting tensile strength of shotcrete with different fiber dosages reached 6.26 MPa, 6.35 MPa, 6.48 MPa, and 5.16 MPa, respectively. This improvement is attributed to continued hydration facilitated by the chloride solution during the early stage of erosion, where newly formed hydration products fill the pore spaces, temporarily enhancing the internal structure of the concrete. As the erosion age increases, the splitting tensile strength of hybrid fiber shotcrete gradually decreases. However, the reduction rate is lower than that observed in conventional shotcrete. This behavior is attributed to the progressive diffusion of chloride ions into the concrete, forming expansive hydration products that damage the internal structure. The presence of dispersed fibers helps to mitigate structural deterioration, thereby enhancing the durability of hybrid fiber shotcrete. As shown in Figure 5b, the increase in splitting tensile strength of shotcrete for 28 d of chloride salt erosion in the ocean atmosphere is smaller than that in the ocean underwater zone. The reason is that the ocean atmosphere’s wet–dry cycle has insufficient water supply, which hinders the further hydration of the concrete and prevents the formation of a practical strength compensation effect as in the ocean underwater zone. Although the hybrid fiber shotcrete in group A3 exhibited the highest splitting tensile strength, its performance under salt spray erosion declined by 4.96%, 14.40%, and 28.64% at 56 d, 84 d, and 112 d, respectively, compared to the 28 d result. This reduction is attributed to the alternating wet–dry cycles in the marine atmospheric environment, which promote repeated salt crystallization on the concrete surface, exacerbating microcrack propagation and accelerating strength degradation.

#### 3.1.3. Flexural Strength Analysis

**Figure 6:**
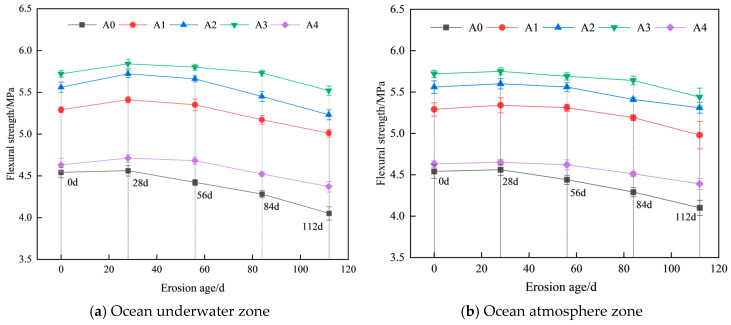
Variation in flexural strength of hybrid fiber shotcrete.

Based on the test results, graphs depicting the variation in the flexural strength of hybrid fiber shotcrete at different chloride erosion ages in the ocean underwater and atmosphere zones were plotted (Figure 6).

As shown in Figure 6, the 28 d flexural strength of hybrid fiber shotcrete, before exposure to chloride ion erosion, was enhanced by 16.52%, 22.47%, 26.0%, and 1.98%, respectively, compared to conventional shotcrete. Among these, the 0.1% PF and 0.2% BF hybrid mix most effectively enhances shotcrete’s flexural strength. This improvement is attributed to BF’s high modulus of elasticity, which can withstand larger tensile forces, while the small amount of uniformly dispersed PF fills voids and reduces internal defects in the shotcrete. As shown in Figure 6a, the flexural strength of hybrid fiber shotcrete exhibits a trend similar to that of compressive and splitting tensile strength under chloride salt erosion in the ocean underwater zone. However, after 28 d of chloride salt erosion, the increase in flexural strength is the smallest among the three strength indicators. As shown in Figure 6b, the change in flexural strength of hybrid fiber shotcrete under chloride salt erosion in the ocean atmosphere is smaller than in the underwater zone. After 112 d of salt spray erosion, the flexural strength of hybrid fiber shotcrete decreased by 5.29%, 5.94%, 3.5%, and 5.62%, respectively, compared to the pre-erosion values. This is due to the increased penetration of chloride salts into the concrete with prolonged erosion, which accelerates fiber degradation and diminishes the reinforcing effect of the hybrid fibers. However, salt spray erosion is mainly manifested in the deterioration of the concrete surface layer, and the hybrid fibers can still play the role of diaspora association so that the flexural strength of shotcrete can be maintained.

### 3.2. Effects of Different Erosion Environments on the Durability of Shotcrete

#### 3.2.1. Mass Loss Rate Analysis

**Figure 7:**
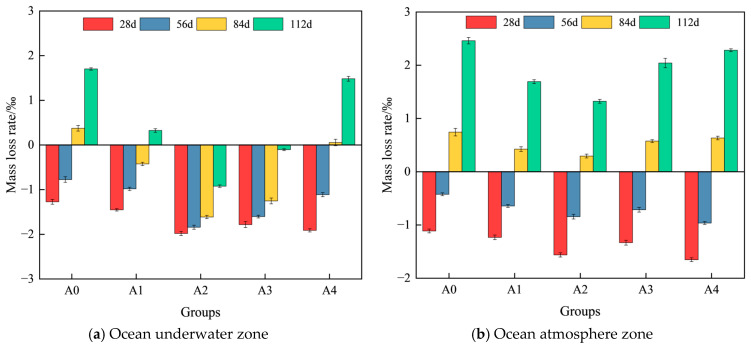
Variation in mass loss rate of hybrid fiber shotcrete.

Based on the test results, the variation in the mass loss rate of hybrid fiber shotcrete at different chloride erosion ages in the ocean underwater and atmosphere zones is shown in Figure 7.

As shown in Figure 7, the mass loss rate of conventional shotcrete is higher than that of hybrid fiber shotcrete at the same erosion age, and the mass loss rate of shotcrete in the ocean atmosphere is greater than that in the ocean underwater. At 112 d of chloride salt erosion, the conventional shotcrete mass loss rate in the ocean atmosphere was approximately 1.45 times that in the underwater zone. This is attributed to the greater variability in temperature and humidity in the ocean atmosphere, where elevated temperatures accelerate chloride ion diffusion and humidity fluctuations induce repeated crystallization of chloride salts. The combined effect is more detrimental than the single high-humidity condition in the underwater ocean. As shown in Figure 7a, the mass loss rate of shotcrete in the ocean underwater zone initially decreases and then increases with the progression of chloride salt erosion. When the age of erosion was 28 d, the mass of hybrid fiber shotcrete increased by 1.46‰, 1.99‰, 1.79‰ and 1.92‰, respectively, compared to the pre-erosion period. This is because the three-dimensional mesh structure formed by hybrid fibers slows the migration of chloride ions, allowing some chloride salt crystals and erosion products to fill microcracks and pores. Meanwhile, unsaturated pores within the concrete absorb part of the chloride salt solution, resulting in a temporary increase in the mass of the shotcrete. As shown in Figure 7b, the trend of shotcrete’s mass loss rate in the ocean atmospheric zone is similar to that observed in the ocean underwater zone. When the erosion age was 112 d, the mass loss rate of hybrid fiber shotcrete decreased by 1.69‰, 1.32‰, 2.04‰ and 2.28‰, respectively, compared with that before erosion. The reason is that the deterioration of the concrete surface layer is more serious under wet–dry cycles, and the localized spalling of the surface layer leads to a rapid increase in the mass loss rate.

#### 3.2.2. Relative Dynamic Elastic Modulus Analysis

**Figure 8:**
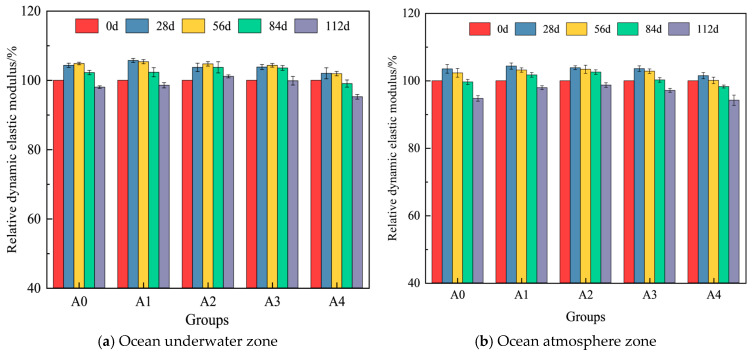
Variation in relative dynamic modulus of elasticity of hybrid fiber shotcrete.

Based on the test results, the variation in the relative dynamic elastic modulus of hybrid fiber shotcrete at different chloride erosion ages in the ocean underwater and ocean atmosphere zones is shown in Figure 8.

As shown in Figure 8, shotcrete’s relative dynamic elastic modulus initially increases and then decreases with the extension of erosion age. The incorporation of an appropriate amount of hybrid fibers enhances shotcrete’s resistance to chloride-induced erosion. At 112 d of immersion and salt spray erosion, the relative dynamic elastic modulus of A2 group hybrid fiber shotcrete was 3.1% and 4.97% higher than conventional shotcrete. However, an excessive amount of hybrid fibers can adversely affect the chloride erosion resistance of shotcrete. This is because an excessive fiber content may reduce the workability of shotcrete, leading to internal defects during casting. Additionally, a high fiber dosage increases the likelihood of fiber agglomeration, weakening the hybrid fibers’ synergistic reinforcing effect and ultimately reducing the shotcrete’s chloride erosion resistance. As shown in Figure 8a, hybrid fiber shotcrete’s relative dynamic modulus of elasticity ranged from 102.03% to 105.75% at 28 d and from 95.24% to 101.13% at 112 d of immersion erosion. Among the mixtures, the 0.1% PF and 0.1% BF blends were most effective in enhancing the shotcrete’s dynamic modulus of elasticity in the early stages. The 0.2% PF and 0.1% BF blend demonstrated better resistance to chloride salt erosion in the later stages. The reason is that PF exhibits good dispersibility and chemical erosion resistance. Increasing its dosage helps fill the concrete’s pores, improves the shotcrete’s density, and enhances its resistance to chloride salt erosion in the later stages. As shown in Figure 8b, salt spray erosion significantly affects the dynamic elastic modulus of shotcrete. After 112 d of salt spray erosion, hybrid fiber shotcrete’s relative dynamic elastic modulus was 97.97%, 98.73%, 97.16%, and 94.22%, respectively. This is because PF’s hydrophobicity is reduced due to salt adsorption in alternating dry and wet environments, diminishing its ability to arrest cracks. Additionally, the alternating dry and wet conditions increase the diffusion rate of chloride ions, leading to a greater depth of chloride salt erosion and, consequently, a decrease in the shotcrete’s relative dynamic modulus of elasticity.

#### 3.2.3. Fine Pore Analysis

**Figure 9:**
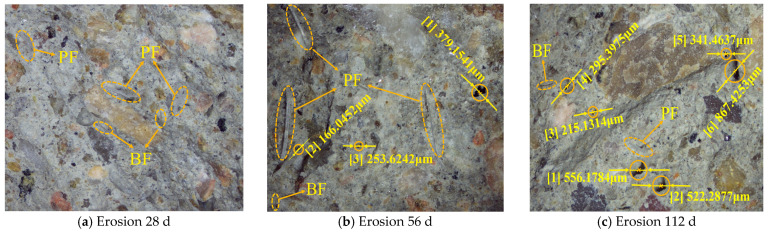
Fine-scale pore distribution in the ocean underwater zone.

**Figure 10:**
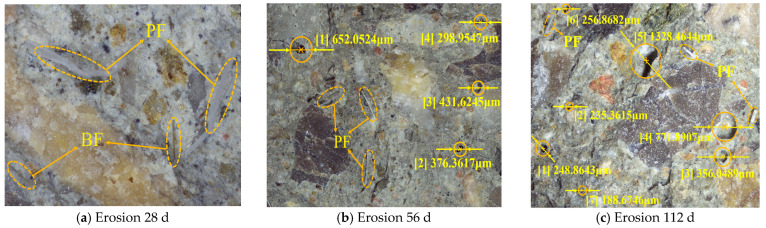
Fine-scale pore distribution in the ocean atmosphere zone.

To investigate the effect of chloride ion erosion on the density of hybrid fiber shotcrete, 3D-DM was employed to examine the microscopic pore distribution at various erosion ages of the A2 group shotcrete. As shown in Figure 9 and Figure 10.

As shown in Figure 9 and Figure 10, after 28 d of chloride salt erosion, the hybrid fiber shotcrete demonstrated excellent compactness in both the ocean underwater and ocean atmosphere zones, with no visible pores observed on the concrete surface. The reason is that an appropriate amount of hybrid fiber improves workability, reduces the number of air voids and pores inside the concrete, refines the internal pore structure, and enhances the compactness of the shotcrete. Additionally, hybrid fibers interweave into a three-dimensional mesh structure, forming a physical barrier to capillary pores, which significantly reduces microcracks’ connectivity and inhibits macrocracks’ propagation. After 56 d of chloride salt erosion, the number of pores on the surface of the hybrid fiber shotcrete increased. However, the number and diameter of the pores were significantly higher on the shotcrete surface in the ocean atmosphere zone than in the ocean underwater zone. The reason is that the ocean’s underwater zone’s erosion environment is more stable, providing sufficient water for further hydration during the middle stage of erosion. This allows hydration products to fill some of the pores. In contrast, the alternating wet and dry conditions in the ocean atmosphere zone cause chloride salts to crystallize repeatedly on the concrete surface, with the expansion stress from these crystals accelerating the development of micropores. After 112 d of chloride salt erosion, the number of pores on the surface of the hybrid fiber shotcrete increased further. The surface porosity of shotcrete in the ocean’s underwater zone exhibits small pore sizes but greater depth due to the saturation of the chloride ion concentration inside the concrete, which causes the breakdown of some hydration products and increases porosity. The pores on the shotcrete surface in the ocean atmosphere zone exhibited larger pore sizes but shallower depths due to the more severe degradation of the concrete surface by salt spray erosion, which caused localized detachment of the top layer of shotcrete.

### 3.3. Effects of Different Erosion Environments on the Microstructure of Shotcrete

#### 3.3.1. XRD Analysis

**Figure 11:**
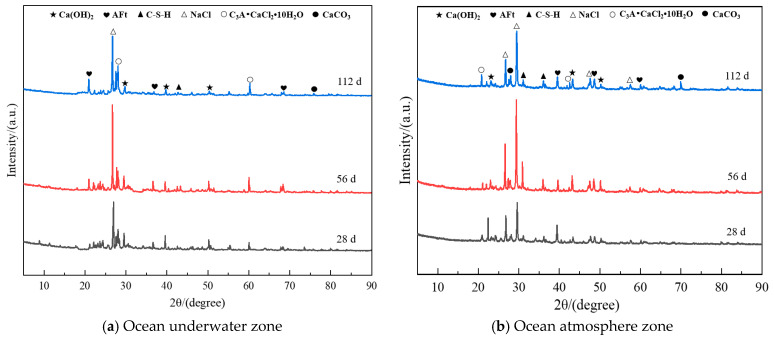
XRD patterns of shotcrete with hybrid fibers at different erosion ages.

To investigate the effect of different chloride ion erosion environments on the hydration products of hybrid fiber shotcrete, the changes in erosion products at various erosion ages of the A2 group shotcrete were analyzed using XRD, as shown in Figure 11.

As shown in Figure 11, the primary hydration products of hybrid fiber shotcrete at different erosion ages include Ca(OH)_2_, AFt, C-S-H, NaCl, CaCO_3_, and C_3_A·CaCl_2_·10H_2_O. Among these, Ca(OH)_2_, AFt, and C-S-H are cement hydration products. NaCl results from the crystallization of chloride salt solution. CaCO_3_ is formed through the carbonation of concrete. C_3_A·CaCl_2_·10H_2_O is a chemical erosion product generated by the reaction of chloride ions with hydration products in the concrete. At 28 d of chloride salt erosion, C_3_A·CaCl_2_·10H_2_O was detected in hybrid fiber shotcrete in both chloride salt erosion environments, with a higher diffraction peak observed in the ocean underwater zone compared to the ocean atmosphere zone. The reason for this is that immersion erosion allows chloride ions to rapidly penetrate the interior of the concrete, leading to the formation of C_3_A·CaCl_2_·10H_2_O from the cement hydration product C_3_A, whereas a small amount of C_3_A·CaCl_2_·10H_2_O primarily forms on the surface of the concrete during the pre-salt spray erosion stage. At 56 d of chloride salt erosion, prominent NaCl diffraction peaks were observed in the hybrid fiber shotcrete, while weak CaCO_3_ diffraction peaks were detected in the shotcrete from the ocean atmosphere zone. This is because the cyclic wet–dry conditions in the ocean atmosphere promote repeated salt crystallization, which accelerates the shotcrete’s surface deterioration. In addition, exposure to air increases the likelihood of contact with CO_2_, leading to the formation of CaCO_3_. At 112 d of chloride salt erosion, the diffraction peaks of Ca(OH)_2_ and C-S-H in the hybrid fiber shotcrete were significantly reduced in both erosion environments, while the CaCO_3_ peak in the shotcrete from the ocean atmosphere zone showed a further increase. The reason is that as the erosion age increases, the crack-arresting ability of hybrid fibers decreases, leading to greater deterioration of the concrete structure. This results in the rapid consumption of Ca(OH)_2_ and C-S-H to form CaCO_3_.

#### 3.3.2. SEM Analysis

**Figure 12:**
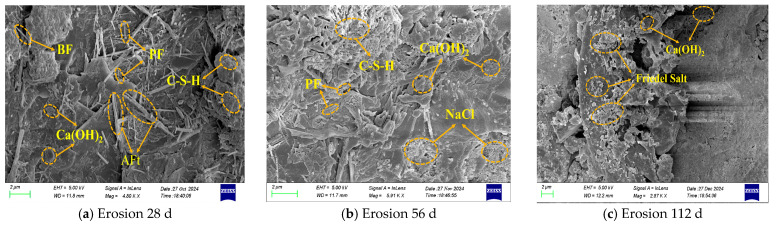
SEM imaging of hybrid fiber shotcrete in ocean underwater zone.

**Figure 13:**
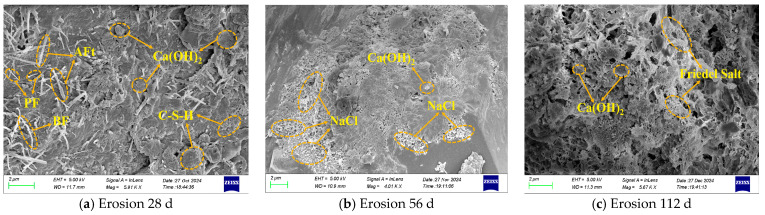
SEM imaging of hybrid fiber shotcrete in ocean atmosphere zone.

To investigate the effect of different chloride ion erosion environments on the microscopic morphology of hybrid fiber shotcrete, the morphology at various erosion ages was observed using a ZEISS Sigma 500 SEM, as shown in Figure 12 and Figure 13.

As shown in Figure 12 and Figure 13, hybrid fiber shotcrete has different microscopic morphologies in different erosion environments. At 28 d of chloride salt erosion, many hydration products appeared within the hybrid fiber shotcrete, primarily C-S-H gels and Ca(OH)_2_ crystals, with a small quantity of AFt interspersed between the C-S-H gels. The three-dimensional mesh structure formed by the hybrid fibers effectively inhibited crack expansion, and a significant amount of hydration products briefly filled the pores of the shotcrete. This phenomenon explains the temporary enhancement in the strength index during the initial period of erosion of hybrid fiber shotcrete. However, the number and size of hydration products in hybrid fiber shotcrete are significantly larger in the ocean underwater zone than in the ocean atmosphere zone. This is because, at the onset of chloride salt erosion, the hybrid fiber shotcrete is still undergoing hydration, and the stable temperature and humidity in the ocean underwater zone provide sufficient water for the hydration process. At 56 d of chloride salt erosion, the hydration products, including C-S-H gel and Ca(OH)_2_ crystals, in hybrid fiber shotcrete were gradually consumed and dissolved. Simultaneously, a large number of NaCl crystals precipitated within the shotcrete in the ocean atmosphere zone, leading to the formation of microcracks in localized areas and a significant increase in porosity. The reason is that with the increase in erosion age, the chloride salt solution penetrates the interior of the shotcrete, reacting with the cement hydration products to generate Friedel’s salt, which causes expansion. Additionally, the chloride salt solution reduces the interfacial bonding properties of the hybrid fibers, thereby decreasing the shotcrete’s fracture-resisting ability. In particular, the alternating dry–wet conditions in the ocean atmosphere zone cause the chloride salts to dissolve and recrystallize repeatedly, forming NaCl crystals. This results in more severe surface deterioration and a higher concentration of internal erosion products in the shotcrete than in the ocean underwater zone. This phenomenon is consistent with the XRD test results. Further, it explains why the shotcrete in the ocean atmosphere zone experiences more severe erosion than in the underwater ocean zone. At 112 d of chloride salt erosion, the cement hydration products in the hybrid fiber shotcrete were significantly degraded; chloride ions caused decalcification of the C-S-H gel, resulting in structural damage, increased internal porosity, a more porous microstructure, and intensified microcrack development. This phenomenon accounts for the rapid decline in shotcrete’s strength and durability during the later stages of chloride salt erosion.

### 3.4. Discussion

#### 3.4.1. Analysis of Hybrid Fiber Distribution Characteristics

**Figure 14:**
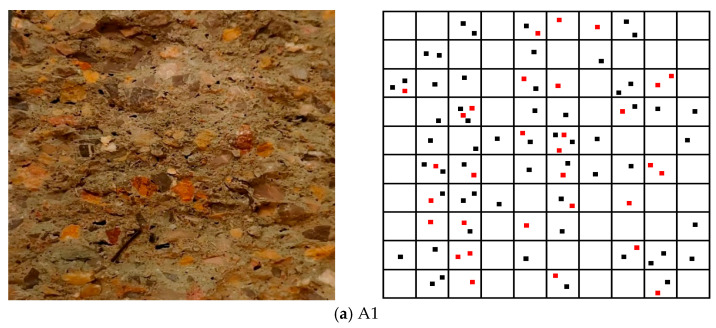

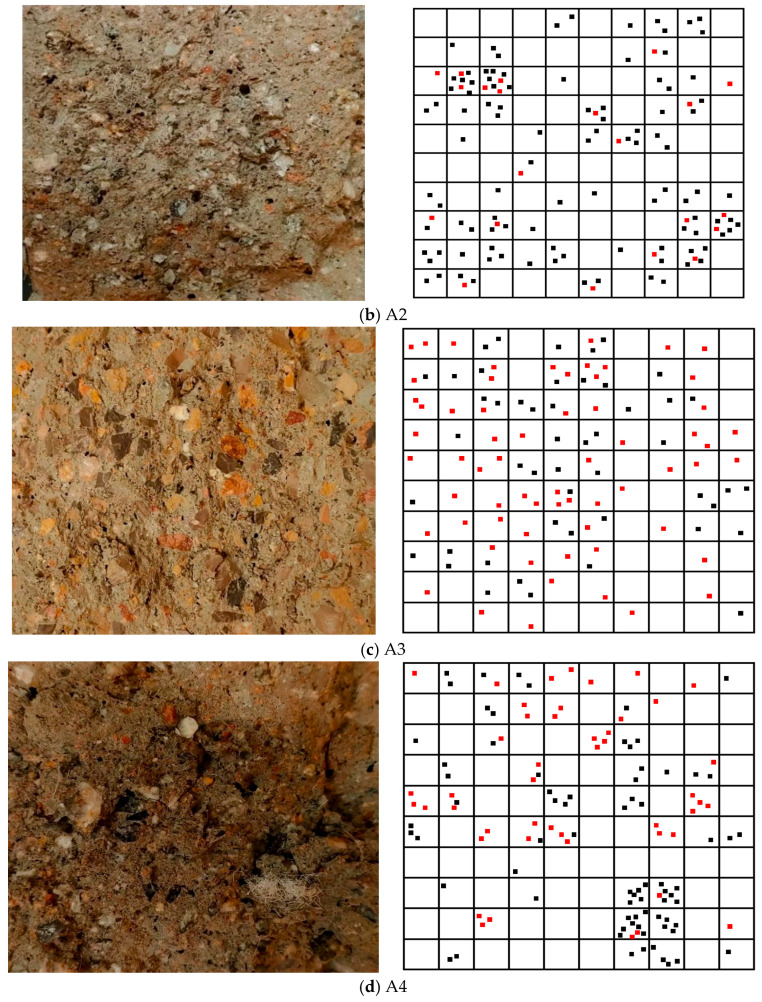
Characteristics of hybrid fiber distribution.

To further analyze the influence of the PF and BF mixing ratio on shotcrete performance, a distribution diagram of mixed fibers at different dosages was created based on the splitting surface of the shotcrete specimen. In the diagram, black dots represent PF and red dots represent BF, as shown in Figure 14.

As shown in Figure 14, the distribution of hybrid fibers on the splitting surface of shotcrete appears random; however, PF and BF exhibit distinct distribution patterns depending on their mixing ratios. As shown in Figure 14a, the dispersion of hybrid fibers in shotcrete is more uniform at lower fiber dosages. However, the low degree of interweaving between PF and BF hinders the formation of a spatial mesh structure. This observation helps explain why group A1 shows limited improvement in shotcrete performance. As shown in Figure 14b,c, the fiber dispersion rate decreases compared to group A1 as the PF and BF mixing ratio increases. However, the degree of interweaving between the two fiber types improves, significantly increasing shotcrete strength. In group A3, fiber dispersion remains relatively high, likely due to BF’s smaller length and diameter, which allows them to disperse more easily during mixing. As shown in Figure 14d, the dispersion rate of hybrid fibers in shotcrete decreases sharply with a continued increase in the fiber mixing ratio, and fiber agglomeration is observed. This is because fiber dispersion in shotcrete is influenced by fiber content, mixing time, and construction wind pressure, with fiber content having the most significant impact [62,63]. This explains why the mechanical properties and durability of shotcrete in group A4 are lower than in the other groups.

#### 3.4.2. Analysis of Socioeconomic Benefits

Hybrid fiber-reinforced shotcrete demonstrates significant economic and environmental benefits in ocean engineering applications. From an economic perspective, enhancing the resistance of shotcrete to chloride ion penetration can effectively prolong the service life of structures such as subsea tunnels, port wharves, and coastal protection systems. The results of this study indicate that hybrid fibers can significantly reduce strength degradation and mass loss under chloride attack, thereby decreasing maintenance frequency and lowering life-cycle costs. In addition, compared with conventional steel fiber-reinforced shotcrete, PF and BF, as non-metallic fibers, can effectively avoid the corrosion issues associated with steel fibers, while offering lower material costs and better construction adaptability. From an environmental perspective, improved durability contributes to reduced resource consumption throughout the entire life cycle of the structure. Moreover, non-metallic fibers such as PF and BF do not produce corrosion by-products, resulting in minimal environmental impact. The reduction in maintenance and reconstruction activities further leads to lower energy consumption and carbon emissions, aligning with the current demands for low-carbon and sustainable development in engineering practice.

#### 3.4.3. Limitations and Future Research Directions

Although this study analyzed the chloride ion resistance of blended-fiber shotcrete under various erosion conditions, it still has some limitations. Due to limitations in experimental conditions, this study was conducted under simulated laboratory conditions and did not take into account complex marine factors, such as the effects of loading and multi-ion interactions on the properties of hybrid fibers. Therefore, field tests in a ocean environment will be required in the future to verify the resistance of hybrid fiber-reinforced shotcrete to chloride corrosion.

## 4. Conclusions

This study incorporated PF and BF to optimize shotcrete mix proportions and evaluate their performance. The chloride salt erosion environments of the ocean underwater and atmosphere zones were simulated through immersion and salt spray tests to analyze the effects of different erosion conditions on the mechanical properties and durability of hybrid fiber shotcrete. Based on the results of 3D-DM, XRD, and SEM analyses, the deterioration mechanism of hybrid fiber shotcrete under chloride ion erosion was investigated from both microstructural and microscopic perspectives. Based on the presented research, the following conclusions can be drawn:

(1) Hybrid fibers significantly enhance the mechanical properties of shotcrete. At 28 d, the compressive strength, splitting tensile strength, and flexural strength of hybrid fiber shotcrete increased by 3.29–18.89%, 3.58–28.03%, and 1.98–26.00%, respectively, compared to conventional shotcrete.
(2) Hybrid fibers can reduce the mass loss and the degradation of relative dynamic elastic modulus in shotcrete subjected to chloride salt erosion. At 112 d of immersion and salt spray erosion, the relative dynamic elastic modulus of shotcrete containing 0.2% PF and 0.1% BF increased by 3.1% and 4.97%, respectively, compared to conventional shotcrete.
(3) Shotcrete demonstrated good resistance to chloride salt erosion in both the ocean underwater and atmosphere zones when the volume fractions of PF and BF were 0.2% and 0.1%, respectively.
(4) The deterioration of shotcrete is more severe in the ocean atmosphere zone than in the ocean underwater zone. In the underwater zone, chloride salt erosion is primarily driven by chemical corrosion, whereas in the atmosphere zone, it is mainly caused by physical stripping.

## Figures and Tables

**Table 1:** Main chemical components of cement.

Element	SiO_2_	CaO	Al_2_O_3_	Fe_2_O_3_	MgO	SO_3_	Loss
Content wt/%	22.13	62.41	4.75	3.62	2.15	2.01	1.74

**Table 2:** Main performance index of fiber.

Types	Density/(g·cm^−3^)	Diameter/μm	Length/mm	Elongation/%	Tensile Strength/MPa	Elastic Modulus/GPa
BF	2.65	17	6	3.01	1256	76.1
PF	0.91	25	12	20.00	510	5.5

**Table 3:** Volume ratio of mixed fibers in different mix ratios.

Number	PF Volume Ratio/%	BF Volume Ratio/%
A0	0	0
A1	0.1	0.1
A2	0.2	0.1
A3	0.1	0.2
A4	0.2	0.2

**Table 4:** Slump test results.

Number	A0	A1	A2	A3	A4
Slump value/mm	155	140	162	132	108

## Data Availability

The original contributions presented in this study are included in the article. Further inquiries can be directed to the corresponding author.
